# Positron Emission Tomography Combined With Computed Tomography as a Screening Tool for Occult Malignancy in Patients With Unprovoked Venous Thromboembolism: An Observational Study

**DOI:** 10.1097/MD.0000000000000110

**Published:** 2014-10-31

**Authors:** Maria Chauchard, Khadija Benali, Thomas Papo, Karim Sacre

**Affiliations:** Department of Internal Medicine (MC, TP, KS); Department of Nuclear Medicine (KBA), Bichat Hospital, Assistance Publique Hôpitaux de Paris; INSERM U1149 (TP, KS), University Paris Diderot, PRES Sorbonne Paris Cité; and DHU FIRE (Fibrosis, Inflammation and Remodelling in Renal and Respiratory Diseases) (TP, KS), Paris, France.

## Abstract

Venous thromboembolism (VTE) can be the first clinical manifestation of an occult malignancy. We aimed to assess the value, in daily practice, of positron emission tomography combined with computed tomography (PET-CT) for occult malignancy diagnosis in patients with unprovoked VTE.

All PET-CTs performed over 5-years period (from January 2009 to October 2013) in adult patients followed in the Department of Internal Medicine (Bichat Hospital, Paris, France) were retrospectively reviewed. Clinical history, imaging findings, and additional diagnostic tests performed because of PET-CT findings were analyzed.

From January 2009 to October 2013, PET-CT was performed for malignancy diagnosis in 67 consecutive patients with unprovoked VTE. Seventeen patients were excluded because of congenital or acquired thrombophilia, known cancer, estrogen use, inability to confirm VTE diagnosis, or missing data. Fifty patients (25 women; mean age, 65.2 ± 15.9 years) were included. VTE was a first episode in 84% of cases. In 22 (44%) patients, PET-CT showed increased uptake suspicious for malignancy. After additional procedures, malignancy was confirmed in 12/22 patients. In all cases of confirmed malignancies, conventional computed tomography scan (CT-scan) had similar diagnosis yield, as compared with PET-CT. In 10/22 cases, the suspected diagnosis of malignancy could not be confirmed despite extensive workup including specialist visits (n = 5), magnetic resonance imaging (n = 4), gastrointestinal tract endoscopy (n = 3), endometrial biopsies (n = 2), and hysterectomy (n = 1). The cost of additional diagnosis procedures performed because of false positive PET-CT amounted to є1956/patient. Interestingly, considering CT-scan findings only, no further investigation would have been scheduled. No patient with negative or false positive PET-CT was diagnosed with cancer during a mean follow-up of 22 ± 13.6 months.

A diagnosis strategy based on PET-CT screening for malignancy in patients with unprovoked VTE had limited diagnosis value and may lead to unnecessary alarming and money- and time-consuming investigations.

## INTRODUCTION

Cancer patients have an increased risk of venous thromboembolism (VTE). Conversely, VTE may be the first clinical manifestation of an occult malignancy.^1^ Because early diagnosis might imply a better prognosis, the search for hidden cancer in patients with unprovoked VTE is warranted. However, no optimal screening strategy for occult cancer in patients with unprovoked VTE has been established yet.^2^

The 2-[F-18] fluoro-2-deoxy-d-glucose (FDG) positron emission tomography combined with computed tomography (PET-CT) is a noninvasive technique routinely used for the diagnosis and staging of malignancies.^3^ To date, only 3 studies have evaluated the interest of PET-CT as a screening tool for occult malignancy in patients with unprovoked VTE.^4–6^ All studies showed that PET-CT screening to detect occult malignancy displayed high sensitivity and negative predictive values. However, PET-CT was performed as a single tool and no direct comparison with conventional computed tomography scan (CT-scan) was performed. Moreover, despite a low positive predictive value (PPV) of PET-CT screening in all studies, the extent of unnecessary additional explorations triggered by false positive PET-CT has not been analyzed.

The aim of our study was to determine the value of PET-CT as a tool for occult malignancy diagnosis in patients with an unprovoked VTE. Specifically, we analyzed the additive diagnosis value of PET-CT as compared with conventional imaging and focused on the nature and costs of additional diagnosis explorations performed because of PET-CT findings.

## METHODS

### Patients

All PET-CTs performed over a 5-years period (from January 2009 to October 2013) in adult patients followed in the Department of Internal Medicine (Bichat Hospital, Paris, France) were retrospectively reviewed. Among them, patients in whom PET-CT was performed to screen for occult malignancy in a context of unprovoked VTE were included. Clinical history, imaging findings including conventional CT-scan, and all additional diagnosis tests performed because of PET-CT findings and final diagnosis were collected from medical records and analyzed.

Unprovoked VTE was defined as VTE occurring in the absence of known cancer, trauma, surgery, or immobilization (≥3 days) in the previous 3 months, pregnancy or postpartum period, or travel >6 hours in the previous month.

All VTE diagnoses were objectively confirmed by Doppler ultrasound for deep vein thrombosis (DVT) and CT-scan and/or ventilation/perfusion lung scan for pulmonary embolism (PE).

Occult malignancy was defined as histology-confirmed malignancy not known to be present at the time of VTE diagnosis.

All patients <50 years were also screened for thrombophilia including the search for antithrombin, protein C, and protein S deficiencies, activated protein C resistance, prothrombin gene mutation (G-20210-A), and antiphospholipid antibodies. Exclusion criteria included known cancer, congenital or acquired thrombophilia, estrogen use, inability to confirm VTE diagnoses, and incomplete information.

### PET-CT Imaging

18-FDG PET-CTs were performed using a combined PET-CT scanner (Discovery 690; GE Healthcare, CT) after an overnight fast. Images were obtained from the skull base to the proximal thighs (3 minutes/bed position), 60 minutes after intravenous administration of 4 MBq/kg 18F-FDG. In diabetic patients, glucose should be <12.0 mmol/L at the time of 18-FDG injection. Low-dose CT (100 keV and 140 mA with current modulation system) without contrast enhancement was acquired for anatomic correlation and attenuation correction of the PET data. PET images were reconstructed using 3-dimensional time-of-flight ordered subset expectation maximization with and without attenuation correction and reoriented in axial, sagittal, and coronal slices (3 mm cross-section thickness and 256 × 256 matrix for a visual field of view of 60 cm). Reconstructed images were displayed on an Advantage Workstation (GE Healthcare) for visual analysis. PET (attenuation corrected and noncorrected) images alone and coregistered PET-CT images were analyzed by 2 senior nuclear medicine physicians to detect foci of nonphysiological hypermetabolism. The 18-FDG uptake was measured by generating a volume of interest to calculate the maximum standardized uptake value. When appropriate, patients underwent further diagnosis procedures to confirm or rule out malignancy.

### Ethics Statement

Our study is a human noninterventional study where subjects were not assigned to treatment; they were assigned to a diagnosis strategy within the current practice (ie, PET-CT); the study involved products with a marketing authorization that are prescribed in the usual manner and used in accordance with authorizations by French agencies (ie, PET-CT); epidemiological methods were used to analyze the data; information used in the study were collected for clinical care. According to the Public Health French Law (art L 1121-1-1, art L 1121-1-2), written consents are not required for human noninterventional studies. Patients were however informed that data collected in medical records might be used for research study in accordance to privacy rule. The local ethics committee has reviewed and approved the study (IRB 00006477- HUPNVS, Paris 7 University, AP-HP).

## RESULTS

### Patients’ Characteristics

**FIGURE 1:**
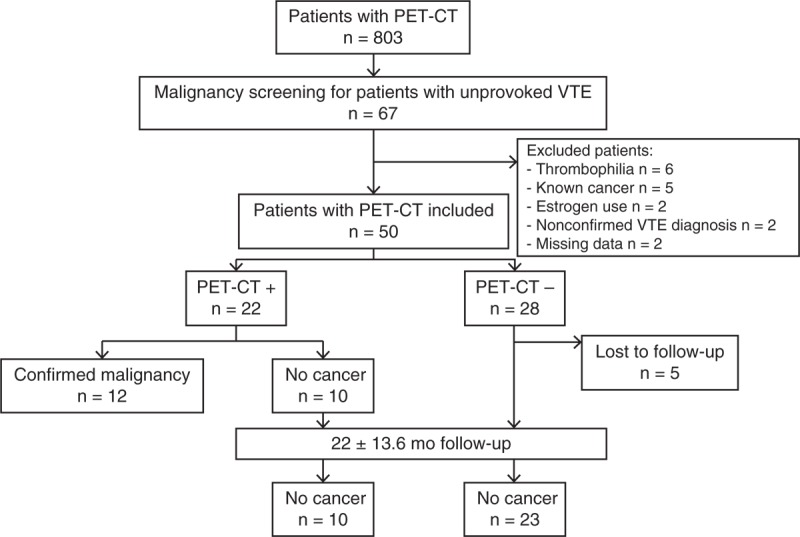
Study patients. PET-CT+ referred to PET-CT showing increased uptake suspicious for malignancy. PET-CT− referred to PET-CT without increased uptake suspicious for malignancy. PET-CT = positron emission tomography combined with computed tomography, VTE = venous thromboembolism.

From January 2009 to October 2013, 803 PET-CTs were performed for clinical purpose in the Department of Internal Medicine (Bichat Hospital). Among them, 67 (8.3%) PET-CTs were performed for malignancy diagnosis in patients with unprovoked VTE. Seventeen patients were excluded because of congenital or acquired thrombophilia (n = 6), known cancer (n = 5), estrogen use (n = 2), inability to confirm VTE diagnosis (n = 2), and missing data (n = 2) (Figure 1).

Fifty patients were included. The average age was 65.2 ± 15.9 years, and 50% of the patients were women. PET-CT was performed between 2 days and 20 weeks after VTE diagnosis (mean, 4.3 ± 4.7 weeks). VTE was a first episode in 84% (42/50) of cases. In 33 cases (66%), patients had a PE with or without DVT. PE was considered massive in 5 cases according to current criteria.^7^ Characteristics of the studied population are given in Table 1.

**TABLE 1:**
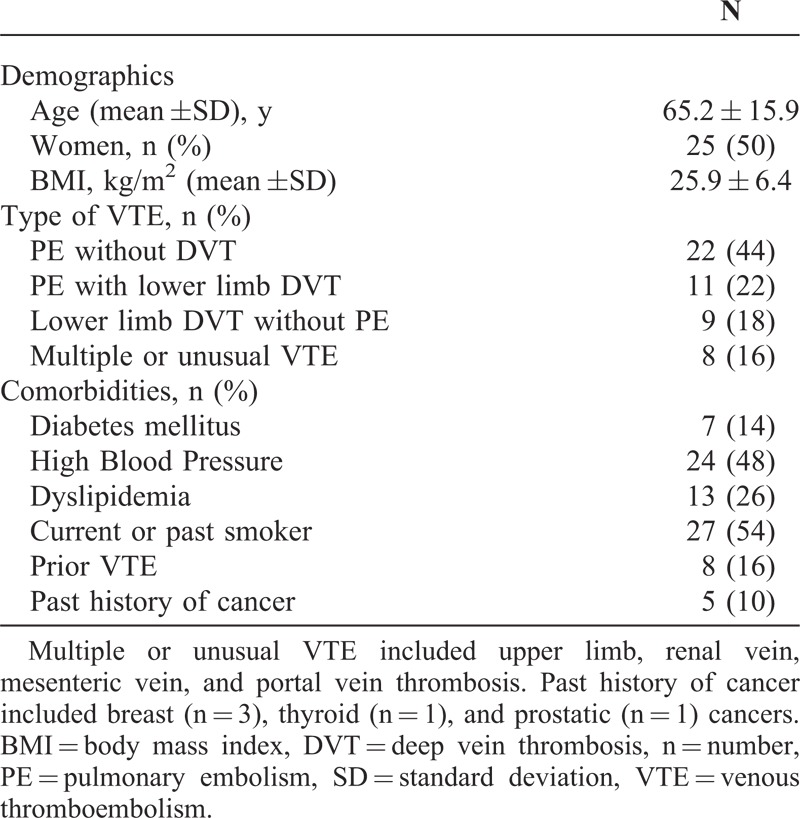
Characteristics of the Patients

### Imaging Data

In 22 patients (44%), PET-CT showed increased FDG-uptake suspicious for malignancy (Table 2). After additional procedures, malignancy was confirmed in 54.5% (12/22) of cases. All malignancies were diagnosed at advanced stages. Six patients (50%) died between 2 and 15 months (median, 3.5 months) after the diagnosis. All 12 patients with malignancy had PE that was massive in 3 cases. Four patients had DVT and 2 patients had unusual VTE, including upper limb and renal vein thrombosis, associated with PE.

**TABLE 2:**
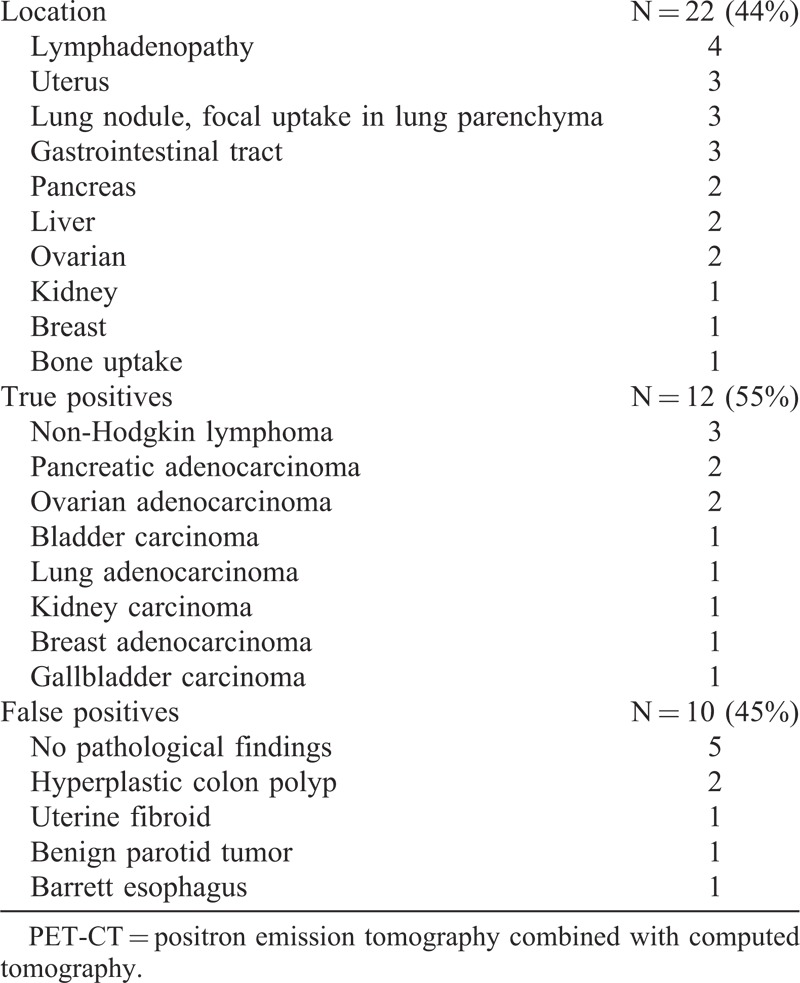
PET-CT With Increased Uptake Suspicious for Occult Malignancy

In all cases of confirmed malignancies, conventional total body CT-scan performed at the same time had equivalent diagnosis accuracy as compared with PET-CT.

In 45.5% (10/22) of cases, additional evaluation, including specialist visits (n = 5), magnetic resonance imaging (n = 4), gastrointestinal tract endoscopy (n = 3), endometrial biopsy (n = 2), and hysterectomy (n = 1), discarded the presence of an occult cancer. The total cost of additional diagnosis procedures performed on patients with false positive PET-CT amounted to є19,559 (є1956/patient). Interestingly, CT-scan was negative in all 10 cases. According to conventional CT-scan findings only, no further investigation would have been scheduled. Eventually, none of the 10 patients with suspicious PET-CT uptakes developed malignancy during the 22 ± 13.6 months follow-up.

Twenty-eight patients (56%) had a negative PET-CT at VTE diagnosis. Five patients were lost to follow-up. Among the 23 other patients, none had developed malignancy at the end of follow-up.

## DISCUSSION

From our study, PET-CT is confirmed to have high negative predictive value for the diagnosis of malignancy in patients with unprovoked VTE. However, PET-CT did not offer higher diagnosis accuracy than conventional CT-scan. PPV of PET-CT screening (PPV = 0.55 in our series) was low. Moreover, unnecessary, costly, time-consuming, and potentially hazardous investigations were performed because of PET-CT positive results. Our findings do not support a search of a hidden cancer strategy based on PET-CT in patients with unprovoked VTE.

The prevalence of cancer in our population was high (24%) and contrasts with the prevalence (range, 0%–7%) reported in previous series.^4–6^ Our study being retrospective, we cannot exclude a systemic bias to explain this apparent discrepancy. However, as probability of hidden cancer increases with age, it should be noticed that our overall population tended to be 10-years older as compared with other series.^5^ Moreover, despite the high prevalence of cancer, PPV of PET-CT screening still appears disappointedly low.

Although controversial, the main objective of an extensive screening for a hidden cancer is to detect more cancers at earlier stages and impact on survival. In our study all cancers revealed by VTE were at advanced stage. Such a high frequency of advanced stage cancer may explain the good diagnosis value of conventional imaging. On the other hand, we may have missed the target population—that is, with cancer diagnosed at early stage—for a systematic PET-CT screening. Some authors tried to improve the selection of candidate patients by using plasma biomarkers known to be involved in the risk of VTE in cancer patients.^6^ In their study, malignancy was confirmed in 7/31 patients (22.6%), with 6 of them at early stage, and addition of plasma biomarker to the PET-CT screening strategy improved the PPV from 0.22 to 0.37.

Our study has several limitations: it is a retrospective analysis of a small number of patients with a relatively short follow-up time.

In conclusion, the use of PET-CT for the screening of occult malignancy in patients with unprovoked VTE has limited diagnosis value and may lead to unnecessary, alarming, and costly investigations in a high proportion of patients.

## ACKNOWLEDGMENT

The authors thank Drs J.F. Alexandra, M.P. Chauveheid, C. Compain, A. Dossier, D. Gobert, C. Gorgiard, O. Lidove, and E. Pasqualoni for their help with patients’ screening. The authors thank Drs S. Burg, H. Hassani, D. Le Guludec, S. Sebahoun, and J. Slama for their excellent support in the interpretation of PET-CT images.
